# A randomized controlled trial comparing sleep hygiene advice with a self-help book focusing on cognitive behavioral therapy for insomnia: a study among patients with prescribed hypnotics from the GP

**DOI:** 10.1080/02813432.2025.2525423

**Published:** 2025-06-27

**Authors:** Bjørn Bjorvatn, Ragnhild Stokke Lundetræ, Øystein Vedaa, Ståle Pallesen, Linn Nyjordet Evanger

**Affiliations:** ^a^ Department of Global Public Health and Primary Care, University of Bergen, Bergen, Norway; ^b^ Norwegian Competence Center for Sleep Disorders, Haukeland University Hospital, Bergen, Norway; ^c^ Department of Health Promotion, Norwegian Institute of Public Health, Bergen, Norway; ^d^ Department of Psychosocial Science, University of Bergen, Bergen, Norway

**Keywords:** Sleep medication, CBT, sleep problems, PraksisNett, insomnia

## Abstract

**Background:**

Chronic insomnia is commonly treated with hypnotics. However, the treatment of choice is cognitive behavioral therapy for insomnia (CBTi). We investigated whether a self-help book based on CBTi is effective in reducing hypnotic use and improving sleep.

**Methods:**

Patients who had received a prescription from their GP for z-hypnotics (zopiclone or zolpidem) in the last 6 months were recruited through PraksisNett, an infrastructure within General Practice, for a randomized controlled trial comparing written materials in form of a sheet of sleep hygiene advice and a self-help book. The participants completed an online questionnaire about hypnotic use, insomnia symptoms, sleep duration, anxiety and depression before the intervention and at 4-5 months follow-up.

**Results:**

In total, 125 patients (response rate 72.7%) completed the follow-up questionnaire. Interaction analyses indicated effects favoring the self-help book for hypnotic use and anxiety. The book reduced the proportion using hypnotics daily from 25.4% to 18.6%, while the proportion increased from 21.2% to 22.7% in the sleep hygiene group. The proportion reporting anxiety was reduced from 32.1% to 23.2% in the self-help book group, while it increased from 27.0% to 31.7% in the sleep hygiene group. Insomnia symptoms were lowered in both intervention groups, whereas depression remained unchanged.

**Conclusion:**

This study indicated that the self-help book was an effective low-threshold treatment option that seems to reduce hypnotic use and at the same time improve sleep and mental health. The patients who received sleep hygiene advice also reported some positive effects, but daily hypnotic use and anxiety increased.

## Introduction

Sleep problems are common in the general population. The most frequent sleep disorder is insomnia, which is characterized by difficulty falling asleep, nocturnal awakenings and/or early morning awakenings, and associated daytime impairments [1,2]. Approximately one in three adults reports occasional sleep difficulties, whereas chronic insomnia is present in 10-20% [1,3,4].

Sleep problems are commonly treated with medications. However, hypnotics are not recommended in patients with chronic insomnia, at least not for daily use, because they can lead to tolerance, dependence and abuse [1,5]. The treatment of choice for chronic insomnia is cognitive behavioral therapy for insomnia (CBTi), which provides larger and longer-lasting effects compared to hypnotics [1,2,6]. A study from Norway shows that among patients using hypnotics, 80% would prefer non-pharmacological treatment to medication, but less than 10% have been offered such treatment [7].

Cognitive behavioral therapy for insomnia comprises various treatment components, of which sleep restriction and stimulus control have the best documented effect [1,8]. Sleep hygiene, cognitive techniques and relaxation exercises are also often part of CBTi.

Sleep hygiene involves basic advice for optimizing sleep. However, such advice, as a stand-alone intervention, is not considered effective for chronic insomnia [1,9]. Sleep restriction is a treatment intended to increase sleep pressure [1]. Many patients with insomnia compensate by spending a long time in bed, hoping to get more sleep. It is relatively common for patients with only 5 h of sleep per night to lie in bed for more than 8 h. This can perpetuate sleep difficulties, as the bed becomes associated with wakefulness. When sleep restriction is initiated, the time in bed is reduced to the time the patient actually sleeps (still not to < 5 h). The rise time is fixed in accordance with the work/school obligations of the patient. During the course of treatment, the time in bed is adjusted depending on sleep efficiency (sleep duration/time in bed * 100%), which is calculated weekly. If sleep efficiency exceeds 80-85%, the patient can go to bed 15 min earlier the following week. If sleep efficiency is lower than 80%, the bedtime remains unchanged [2]. Stimulus control aims at changing sleep-incompatible associations with the sleep environment and ensuring that the patient associates the bed and bedroom with sleep. According to the instructions, the patients should leave their bedroom temporarily if sleep onset has not occurred within a short time (20-30 min). The same recommendation applies to nocturnal awakenings. The bed should not be used for activities other than sleep, i.e. one should not read, watch TV, be on the mobile phone or worry in bed. Sexual activity is allowed. Sleeping during the day is discouraged [1,10]. Cognitive techniques aim to identify, test the validity of, and change thought patterns assumed to maintain the sleep difficulties [1].

The objections to CBTi are that the treatment is complicated, time-consuming, and difficult to access [1,11,12]. Few general practitioners (GPs) offer such treatment, and there are few specialized treatment centers. It has therefore been desirable to make the treatment more accessible [1]. Meta-analyses of self-help therapies for insomnia conclude that such interventions are effective [1]. However, more studies are needed [1,13,14].

We have previously documented the effect of a self-help book based on CBTi in a randomized controlled trial, where the book was compared to sleep hygiene advice [12]. The participants in that study were self-referred *via* newspaper advertisements. In the group that received the self-help book, the proportion reporting the use of sleeping pills (hypnotics) was significantly reduced, while in the group that received sleep hygiene advice, the proportion increased [12]. However, that study comprised relatively few hypnotic users. It is therefore uncertain whether the self-help book will be effective among patients who use hypnotics.

The aim of the current study was to compare the effects of two written materials, that is, a sheet with sleep hygiene advice (typical advice given in clinical practice) and the self-help book, in a randomized controlled trial among patients who have been prescribed hypnotics by their GP. We are not aware of any similar studies, either from Norway or internationally. We had the following hypotheses: 1) The self-help book will reduce the use of hypnotics more than the sleep hygiene advice. 2) The self-help book will improve sleep and mental health more than the sleep hygiene advice.

## Materials and methods

The study was conducted *via* the national infrastructure PraksisNett, where GP practices from all over Norway participate [15]. Using PraksisNett’s privacy-protecting IT system, each participating GP obtained a list of their own patients over the age of 18 who had been prescribed the hypnotics zopiclone or zolpidem within the past 6 months. The GP then invited the patients to the study *via* an electronic message through www.helsenorge.no. A total of 18 GPs participated, and a total of 455 invitations were sent digitally in the period May-November 2023. The invitation letter contained a link to a website (provided by SurveyXact by Ramboll; www.surveyxact.no), where the patients received information about the study and could accept or decline participation. The patients were informed that the study compared two types of written material to improve sleep, but they were not given any details about what type of material. Only patients who consented were given access to the online questionnaire. A total of 197 patients consented (response rate 43.3%). Only 15 patients actively declined to participate, i.e. a total of 212 patients visited the study website. In total, 172 participants provided the contact information necessary to receive the written material and thus constituted the study sample.

The study was a blinded randomized controlled comparative study of two types of written material: 1) A sheet with sleep hygiene advice (Table 1) and 2) the self-help book ‘Better sleep. A handbook for you who sleep poorly’, 2^nd^ edition. The self-help book has 187 pages and was published in the fall of 2013 [16]. The book is written for patients suffering from sleep problems and the readers need to be fluent in Norwegian to benefit. It covers normal sleep and sleep regulation, how sleep problems are assessed, and describes various causes of poor sleep. The main focus of the book is on the treatment of chronic insomnia with CBTi, which is reviewed in detail in the book. To illustrate the therapy in the most concrete and recognizable way possible, a typical patient is followed through the assessment, diagnosis and treatment of the insomnia. The goal is that readers should be able to carry out the treatment program on their own. Participants were randomized consecutively to either sleep hygiene advice or self-help book using an electronic randomization generator (https://www.randomlists.com/team-generator).

**Table 1.** Sleep hygiene advice.

Avoid caffeinated drinks during the last hours before bedtime (coffee, tea, cola) Avoid smoking/nicotine during the last hours before bedtime Avoid alcohol as a sleep aid Avoid going to bed hungry, but do not consume a heavy meal before bed Keep the bedroom dark, quiet and with moderate temperature. If necessary, use mask and earplugs Regular exercise is good, but do not exercise during the last hours before bedtime

After 4-5 months, participants received an email from SurveyXact asking to complete the follow-up questionnaire. Up to two email reminders were sent. If no response was received, the patient received a phone call to check whether the email had been received. Some requests had ended up in spam filters. Participants who completed the follow-up survey were offered the written material that the other group had received. Beyond this, participants received no compensation.

### Questionnaire

The patients provided information about their gender, age, marital status, and educational level. Participants were asked about their use of hypnotics, sleep duration, and how long they had suffered from sleep problems. Participants were also asked about potential comorbid conditions (Table 2).

**Table 2.** Demographics and values on various questions before the intervention among 172 patients who had been prescribed hypnotics by their GP in the past 6 months.

	Total population	Sleep hygiene advice	Self-help book
	(*n* = 172)	(*n* = 86)	(*n* = 86)
Age			
18–35	9 (5.2%)	5 (5.8%)	4 (4.7%)
36–50	35 (20.3%)	15 (17.4%)	20 (23.3%)
51–65	68 (39.5%)	37 (43.0%)	31 (36.0%)
65+	60 (34.9%)	29 (33.7%)	31 (36.0%)
Gender			
Male	49 (28.5%)	25 (29.1%)	24 (27.9%)
Female	119 (69.2%)	59 (68.6%)	60 (69.,8%)
Not answered/other	4 (2.3%)	2 (2.3%)	2 (2.3%)
Marital status			
Single	38 (22.1%)	22 (25.6%)	16 (18.6%)
Married/cohabiting	109 (63.4%)	52 (60.5%)	57 (66.3%)
Divorced/separated	14 (8.1%)	7 (8.1%)	7 (8.1%)
Widow/widower	11 (6.4%)	5 (5.8%)	6 (7.0%)
Country of birth			
Norway	161 (93.6%)	81 (94.2%)	80 (93.0%)
Other countries in Europe	11 (6.4%)	5 (5.8%)	6 (7.0%)
Asia/Africa/America/Oceania	0 (0.0%)	0 (0.0%)	0 (0.0%)
Educational level			
Primary school	28 (16.3%)	13 (15.1%)	15 (17.4%)
High school	55 (32.0%)	32 (37.2%)	23 (26.7%)
University/college	89 (51.7%)	41 (47.7%)	48 (55.8%)
Children living at home			
Yes	31 (18.9%)	12 (14.0%)	19 (22.1%)
No	141 (82.0%)	74 (86.0%)	67 (77.9%)
Shift work			
No	157 (91.3%)	80 (93.0%)	77 (89.5%)
Yes, but not night work	8 (4.7%)	2 (2.3%)	6 (7.0%)
Yes, including night work	7 (4.1%)	4 (4.7%)	3 (3.5%)
Diabetes			
No	159 (92.4%)	82 (95.3%)	77 (89.5%)
Yes	13 (7.6%)	4 (4.7%)	9 (10.5%)
Hypertension			
No	129 (75.0%)	63 (73.3%)	66 (76.7%)
Yes	43 (25.0%)	23 (26.7%)	20 (23.3%)
Cardiovascular disease			
No	152 (88.4%)	79 (91.9%)	73 (84.9%)
Yes	20 (11.6%)	7 (8.1%)	13 (15.1%)
Neurological disease			
No	138 (80.2%)	66 (76.7%)	72 (83.7%)
Yes	34 (19.8%)	20 (23.3%)	14 (16.3%)
Lung disease			
No	154 (89.5%)	76 (88.4%)	78 (90.7%)
Yes	18 (10.5%)	10 (11.6%)	8 (9.3%)
Rheumatoid disease			
No	127 (73.8%)	59 (68.6%)	68 (79.1%)
Yes	45 (26.2%)	27 (31.4%)	18 (20.9%)
Cancer			
No	160 (93.0%)	79 (91.9%)	81 (94.2%)
Yes	12 (7.0%)	7 (8.1%)	5 (5.8%)
Allergy			
No	124 (72.1%)	63 (73.3%)	61 (70.9%)
Yes	48 (27.9%)	23 (26.7%)	25 (29.1%)
Sleep apnea			
No	156 (90.7%)	79 (91.9%)	77 (89.5%)
Yes	16 (9.3%)	7 (8.1%)	9 (10.5%)
Duration of sleep problems			
Do not have sleep problems	4 (2.3%)	1 (1.2%)	3 (3.5%)
Less than 3 months	2 (1.2%)	1 (1.2%)	1 (1.2%)
3 months to 1 year	4 (2.3%)	3 (3.5%)	1 (1.2%)
1 to 5 years	42 (24.4%)	20 (23.3%)	22 (25.6%)
More than 5 years	120 (69.8%)	61 (70.9%)	59 (68.6%)
Prescribed sleep medication use			
Not daily	126 (73.3%)	65 (75.6%)	61 (70.9%)
Daily	46 (26.7%)	21 (24.4%)	25 (29.1%)
Prescribed sleep medication use			
Number of days per week	3.5 (SD = 2.6)	3.3 (SD = 2.6)	3.8 (SD = 2.7)
Insomnia severity (ISI)			
No insomnia (ISI < 10)	29 (16.9%)	17 (19.8%)	12 (14.0%)
Insomnia (ISI 10-28)	143 (83.1%)	69 (80.2%)	74 (86.0%)
Insomnia total score (ISI)	15.1 (SD = 5.3)	15.0 (SD = 5.1)	15.3 (SD = 5.4)
Sleep duration			
Less than 6 h	82 (47.7%)	39 (45.3%)	43 (50.0%)
6 h or more	90 (52.3%)	47 (54.7%)	43 (50.0%)
Sleep duration (in minutes)	342 (SD = 83)	343 (SD = 82)	340 (SD = 84)
Anxiety (PHQ-4)			
No	123 (71.5%)	64 (74.4%)	59 (68.6%)
Yes	49 (28.5%)	22 (25.6%)	27 (31.4%)
Anxiety total score	2.0 (SD = 1.7)	1.9 (SD = 1.6)	2.1 (SD = 1.8)
Depression (PHQ-4)			
No	123 (71.5%)	63 (73.3%)	60 (69.8%)
Yes	49 (28.5%)	23 (26.7%)	26 (30.2%)
Depression total score	2.0 (SD = 1.7)	1.9 (SD = 1.7)	2.0 (SD = 1.7)

ISI: Insomnia Severity Index; PHQ: Patient Health Questionnaire; SD: standard deviation.

The questionnaire contained several validated instruments: The Insomnia Severity Index (ISI) consists of 7 questions about insomnia [17], and is the most widely used insomnia scale. ISI is recommended for use in clinical studies [1]. The scale provides a total score where higher values indicate more insomnia symptoms, but ISI can also be used to categorize symptoms into ‘no insomnia’ (total score < 10) and ‘insomnia’ (total score ≥ 10) [1]. Mental health was assessed using the Patient Health Questionnaire-4 (PHQ-4), a validated questionnaire with 4 questions about anxiety and depressive symptoms in the past two weeks. The questionnaire is widely used, and a score of 3 or higher on the subscales is used as an indicator of possible anxiety or depression [18].

The primary outcome measures were hypnotic use and insomnia severity (as pre-registered in ClinicalTrials), whereas sleep duration, anxiety and depression comprised the secondary outcome measures.

In the follow-up questionnaire, patients were also asked whether they were satisfied with the written material and whether they had followed the advice. Both questions had 5 response alternatives (strongly disagree, slightly disagree, neither agree nor disagree, slightly agree, strongly agree).

### Inclusion and exclusion criteria

Participants had to be at least 18 years old and had been prescribed zopiclone or zolpidem by their GP within the last 6 months. Beyond that, there were no other inclusion or exclusion criteria.

### Ethical assessment

Bjorvatn is the author of the self-help book and receives royalties according to the usual rules. The first author was blinded to group affiliation until the statistical analyses were conducted. The study has been approved by the Regional Committee for Medical and Health Research Ethics (REK-Nord 2021/352618) and pre-registered in ClinicalTrials.gov (NCT05069285).

### Statistics

Power calculation was conducted using G’Power (version 3.1.7) prior to the study [19]. Based on a small to moderate effect size (*d* = 0.30), alpha set at 0.05, power (1-beta) set at 0.80, and the correlation between pre- and post-measurements set at 0.50, a total of 90 people were needed to demonstrate a significant interaction between group (sleep hygiene vs. self-help book) and time (pre vs. post).

Statistical analyses were conducted in SPSS version 28, where paired t-tests for continuous data and McNemar tests for categorical data were used for within-group comparisons of pre- and post-intervention values for the sleep hygiene advice and the self-help book. To examine the interaction effect between time and group, generalized linear mixed models (GLMM) with logistic regression were used for the dichotomous variables and linear mixed models (LMM) for the continuous variables, with random intercept for participant ID. These analyses were performed in RStudio version 2024.09.0 + 375. The analyses were conducted according to the intention-to-treat (ITT) principle to include all participants who were initially randomized. The significance level was set at 0.05.

For the primary outcome (daily hypnotic use) we additionally fitted a GLMM with penalized quasi-likelihood (glmmPQL, MASS) as a sensitivity analysis. Because PQL does not return likelihood-based fit statistics for binary data, the results were not used to assess model fit; rather, they were inspected to see whether an alternative estimation method materially changed the size or precision of the time × group interaction effect reported in the main GLMM. All mixed-effects models were fitted in R with lme4, using glmer() for logistic GLMMs and lmer() for linear mixed models, while the additional sensitivity analysis employed glmmPQL() from MASS. Confidence intervals and odds ratios were extracted with the standard confint() and exp() functions.

## Results

**Figure 1.**
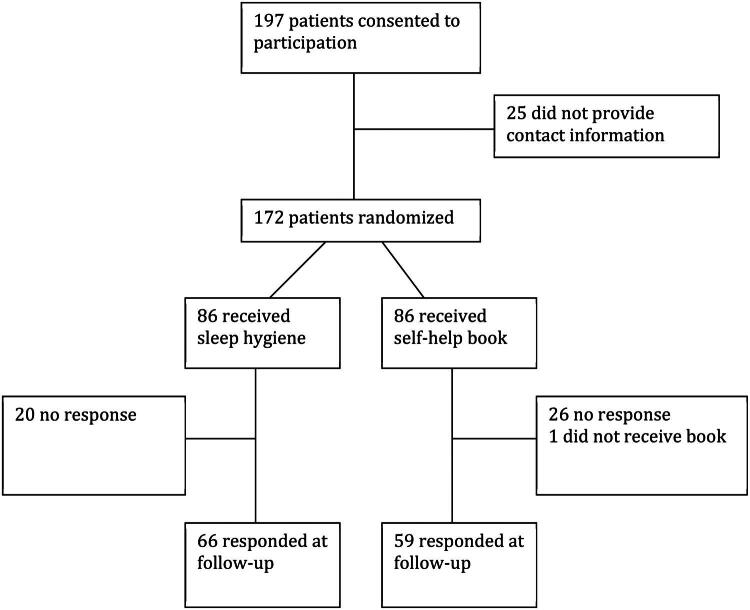
Overview of the participants in the study.

Figure 1 shows an overview of the study. A total of 125 participants completed the follow-up survey, yielding a response rate of 72.7%. Approximately one in four patients reported comorbid somatic diseases such as hypertension, rheumatic disease and allergy (Table 2). Anxiety and depression were reported by 28.5% of the participants. Approximately 70% of the sample reported that the sleep problems had lasted for more than 5 years, while 26.7% reported daily use of prescribed sleep medication. The criteria for insomnia were met by 83.1%.

Table 3 shows data from those who completed the study. The self-help book reduced the proportion of daily hypnotic use from 25.4% to 18.6%, while participants in the sleep hygiene advice group reported a small increase. The interaction effect (time x group) for daily hypnotic use was near significant in the GLMM (*p* = 0.077, Table 3). The GLMM PQL sensitivity analysis yielded a smaller standard error and a narrower 95% confidence interval (CI) for the time × group odds ratio (OR = 6.69, 95% CI = 2.30-19.47, *p* = 0.001) compared with the primary GLMM; however, because PQL lacks likelihood-based fit indices, these results were considered supportive rather than definitive. Both groups had small and non-significant reductions in the proportion with insomnia, and the proportion with sleep duration of 6 h or more increased non-significantly in both groups. There were no interaction effects for these variables. Fewer people reported anxiety in the self-help book group, while the proportion reporting anxiety increased non-significantly in the sleep hygiene group. There was a significant time x group interaction, suggesting positive effects of the self-help book compared with the sleep hygiene advice. The proportion of participants reporting depression remained stable from baseline to follow-up in both groups, with no significant time x group interaction.

**Table 3.** The effects of sleep hygiene advice and self-help book on daily hypnotic use, insomnia, sleep duration ≥6 h, anxiety, and depression among patients who had been prescribed hypnotics by their GP in the past 6 months.

	Sleep hygiene advice, within-group effects				Self-help book, within-group effects				Time x group (GLMM)	
	Pre	Post	McNemar test		Pre	Post	McNemar test		Odds ratio (95%CI)	p-value
Hypnotic use daily			1.000				0.289		37.89 (0.68 − 2118.06)	0.077*
No	52 (78.8%)	51 (77.3%)			44 (74.6%)	48 (81.4%)				
Yes	14 (21.2%)	15 (22.7%)			15 (25.4%)	11 (18.6%)				
ISI			0.424				1.000		0.42 (0.04 − 4.71)	0.485
No insomnia	15 (23.1%)	19 (29.2%)			8 (13.8%)	9 (15.5%)				
(ISI <10)										
Insomnia (ISI 10–28)	50 (76.9%)	46 (70.8%)			50 (86.2%)	49 (84.5%)				
Sleep duration			0.146				0.481		1.15 (0.32 − 4.18)	0.827
<6 hours	27 (41.5%)	21 (32.3%)			26 (44.8%)	22 (37.9%)				
6+ hours	38 (58.5%)	44 (67.7%)			32 (55.2%)	36 (62.1%)				
PHQ-4, anxiety			0.508				0.302		**17.13 (1.33 − 221.24)**	**0.030**
No	46 (73.0%)	43 (68.3%)			38 (67.9%)	43 (76.8%)				
Yes	17 (27.0%)	20 (31.7%)			18 (32.1%)	13 (23.2%)				
PHQ-4, depression			1.000				1.000		1.19 (0.10 − 13.75)	0.891
No	46 (73.0%)	47 (74.6%)			41 (73.2%)	42 (75.0%)				
Yes	17 (27.0%)	16 (25.4%)			15 (26.8%)	14 (25.0%)				

ISI: Insomnia Severity Index; PHQ: Patient Health Questionnaire; CI: confidence interval; GLMM: generalized linear mixed model.

McNemar tests are based on *n* = 63–66 i the sleep hygiene group and *n* = 56–59 in the self-help book group.

*Due to large confidence interval in the GLMM-model and unclear model fit, an additional analysis was conducted using GLMM with penalized quasi-likelihood (PQL) for hypnotic use daily: Odds ratio = **6.69, 95%CI = 2.30-19.47, *p* = 0.001**.

Significant findings are indicated in bold.

Table 4 shows the effects of sleep hygiene advice and the self-help book on the number of days per week with hypnotic use, sleep duration in minutes, and total scores on the insomnia, anxiety, and depression questionnaires. Among those who completed the study, there was a significant reduction in the number of days with hypnotic use only in the group that received the self-help book. The insomnia score improved significantly in both groups. Sleep duration increased significantly in the self-help book group only. There were no changes in the total anxiety and depression scores. We found no significant interaction effects for these variables (Table 4).

**Table 4.** The effects of sleep hygiene advice and self-help book on number of days of hypnotic use, insomnia symptoms, sleep duration, and symptoms of anxiety and depression.

	Sleep hygiene advice			Self-help book			Time x group (LMM)	
	Mean (SD) Pre – Post	t (df), p-value^a^	Cohens d^b^	Mean (SD) Pre – Post	t (df), p-value^a^	Cohens d^b^	B (95%CI), p-value^c^	Cohens d^d^
Hypnotics d/week	3.1 (2.6)−2.8 (2.6)	1.35 (65), 0.181	0.12	3.5 (2.7)−2.8 (2.7)	**3.04 (**58**), 0.004**	0.26	0.45 (0.13 − 1.03), 0.128	0.38
ISI	14.5 (5.2)−13.0 (5.4)	**2.87 (64),0.006**	0.28	15.3 (5.1)−13.4 (5.0)	**3.60 (**57**), <0.001**	0.38	0.31 (−1.14 − 1.75), 0.672	0.11
Sleep duration, min	347 (77)−357 (84)	1.33 (64), 0.190	0.12	343 (84)−365 (97)	**2.61 (**57**), 0.011**	0.24	−12.44. (−34.42 − 9.56), 0.270	−0.28
PHQ-4, anxiety	1.9 (1.5)−2.0 (1.6)	0.51 (62), 0.612	0.06	2.1 (1.7)−1.7 (1.5)	1.82 (55), 0.075	0.25	0.46 (−0.03 − 0.96), 0.066	0.47
PHQ-4, depression	2.0 (1.8)−1.9 (1.6)	0.20 (62), 0.843	0.06	2.0 (1.7)−1.9 (1.5)	0.99 (55), 0.328	0.06	0.13 (−0.31 − 0.57), 0.563	0.15

ISI: Insomnia Severity Index; PHQ-4: Patient Health Questionnaire; SD: standard deviation; df: degrees of freedom; CI: confidence interval.

^a^Paired t-test of values before and after the intervention (separate tests for the group with sleep hygiene and self-help book). In these analyses the number of participants varied from 63 to 66 in the sleep hygiene group and from 56 to 59 in the self-help book group.

^b^Effect size (Cohens d) for paired values.

^c^Linear mixed models (LMM) were conducted to explore the interaction effect between time and group for the continuous outcome variables.

^d^The effect size (Cohens d) for the interaction analyses were calculated by dividing the estimate of the time x group interaction by the pooled standard deviation, here approximated as the residual standard deviation from the model, which represents the variability in the data after adjusting for fixed effects. Significant findings are indicated in bold.

When asked whether participants were satisfied with the written material, 15.9% slightly/strongly disagreed, 47.6% neither nor, 36.5% slightly/strongly agreed in the sleep hygiene advice group, while the corresponding percentages for the self-help book were 0%, 51.8%, and 48.2%, respectively. When asked whether participants had followed the advice, 6.4% slightly/strongly disagreed, 41.3% neither nor, 52.4% slightly/strongly agreed in the sleep hygiene group, while the corresponding percentages for the self-help book group were 1.8%, 39.3%, and 58.9%.

## Discussion

Daily hypnotic use was reduced in the self-help book group, while it increased slightly in the sleep hygiene group, with a time x group interaction effect in favor of the self-help book. Our initial hypothesis, stating that the self-help book would reduce hypnotic use more than the sleep hygiene advice, was thus supported. The self-help book also reduced the proportion of participants with anxiety, while the proportion increased in the group who received the sleep hygiene advice, again with a significant time x group interaction effect. These findings indicate that the self-help book was more effective than the sleep hygiene advice.

The second hypothesis was that the self-help book would improve sleep more than the sleep hygiene advice. We did not find support for this. There was a reduction in the total score on the insomnia scale in both groups. However, sleep duration in minutes was only significantly improved in the self-help book group. Still, none of the interaction analyses for these variables were significant, indicating no different trajectories between the groups. As mentioned above, there was a small increase in the proportion who used hypnotics daily in the sleep hygiene group, which – given that hypnotics have an effect – may have camouflaged a possible interaction effect in favor of the self-help book on both insomnia symptoms and sleep duration. It is also worth emphasizing that even though hypnotic use was reduced for participants receiving the self-help book, sleep improved.

The proportion of anxiety was reduced from 32% to 23% in the self-help book group, while it increased in the sleep hygiene advice group. With a significant time x group interaction effect, the self-help book was evidently better for mental health than the sleep hygiene advice

The study was conducted among patients who received a prescription for z-hypnotics from their GP in the past 6 months. The patients were relatively old, with more than a third being above 65 years of age. Many of the patients had chronic conditions, such as rheumatic diseases, diabetes, cardiovascular diseases, cancer, and sleep apnea. This indicates that the participants belong to a group of patients with heavy comorbidity, and where the GP is often involved. The study therefore reflects this patient group well, where sleep problems are just one of several health challenges.

Surprisingly, only one in four participants reported daily use of hypnotics before the intervention, despite the fact that over 80% met the criteria for insomnia. Such findings may indicate that many GPs have been successful in discouraging daily use of hypnotics, and that many patients follow this advice. Daily use of hypnotics increases the risk of tolerance and dependence, while intermittent use significantly reduces this risk [1].

Despite significant improvements in sleep, many participants still reported insomnia after the intervention. This is common in insomnia treatment studies, both for pharmacological and non-pharmacological interventions [20], and underscores the complex nature of this disorder.

Sleep hygiene advice is often given by doctors in the treatment of sleep problems, but such advice, as a solo intervention, is not considered effective for chronic insomnia [1,9,21]. Current European guidelines therefore conclude that sleep hygiene is not evidence-based treatment [1]. In our study, there were some positive sleep effects of the sleep hygiene advice. This suggests that such advice may be helpful for some patients who are prescribed hypnotics, although it is unclear whether the improvement was anything more than regression to the mean. In addition, the proportion of daily hypnotic use and also the proportion of anxiety increased in the group that received the written sleep hygiene advice, in contrast to the group that received the self-help book. Sleep hygiene advice should therefore be given with caution.

In recent years, there has been a large increase in self-help therapies for insomnia [1]. Several meta-analyses show that CBTi given in the form of self-help books or digital interventions is effective [1]. One problem with such self-help therapies is that the dropout rate during treatment can be high. For online CBTi, the dropout rate can be more than 50% [1]. However, recent developments with CBTi apps for smartphones may lower dropout rates. The response rate after the intervention in our study was 73%, which is relatively high, but lower than what we found in the previous study with the self-help book [12]. The self-help book also had less effect than in the previous study. This was expected, as the previous study only included self-referred participants who met the diagnostic criteria for chronic insomnia, and excluded patients with comorbid conditions such as sleep apnea and restless legs [12]. There was also no requirement to use hypnotics. Patients who use hypnotics are considered a more demanding patient group, and in the clinic many doctors are striving to reduce their prescriptions [22,23]. Effective and scientifically documented self-help therapies have therefore been called for [1,13,24]. There are no other scientifically tested self-help books for insomnia in Norwegian, but a Swedish self-help book has shown good effects among patients with insomnia [25], also 10 years after the intervention [26].

Sleep problems affect many people, and there is a need for easily accessible treatment. In Norway, low-threshold treatment services for insomnia, among other things, are organized according to the mixed or stepped-care model [27]. The lowest level in the stepped-care model is self-administered CBTi provided through, for example, books or the internet, while the highest level encompasses individualized CBTi provided by sleep specialists [13]. European guidelines emphasize that low-threshold therapies must have documented effects before they can be recommended [1]. The self-help book in our study is considered a low-threshold intervention, and the book has documented effectiveness for insomnia [12]. Patients who are not responding to self-help therapies should be offered treatment in-person [13].

### Strengths and limitations

Our study has several strengths and limitations. One strength is that the study was a randomized controlled comparative study of two active treatments. Other strengths were that the response rate was high and that the questionnaires are well validated. A valuable aspect of the study is that we recruited actual patients to receive the self-help material, in contrast to self-referred individuals, which is most common in previous studies. The study had no other inclusion or exclusion criteria than a prescription for z-hypnotics. This increases generalizability. However, the choice of inclusion and exclusion criteria can also be considered a limitation. The patients had many comorbidities, both somatic and psychological. Thus, we cannot disregard that patients who preferably should have received other kinds of treatment were included. The infrastructure PraksisNett was used to recruit patients. It functioned effectively, allowing us to include only patients who had received a prescription for zopiclone or zolpidem in the past 6 months. However, less than half of the patients invited by the GPs agreed to participate. This may indicate that not everyone who uses hypnotics is motivated for self-help based CBTi. Patients who prefer to use hypnotics will therefore presumably participate to a lesser extent. As all communication, from invitation to completing the questionnaire before and after the intervention, was online, only patients who mastered this participated. Another limitation is that people with cognitive impairment will likely not benefit from self-help books focusing on cognitive behavioral therapy. The study is based exclusively on subjective data, and whether sleep was objectively improved is thus not known. There were fewer patients than expected who used hypnotics daily, which created a limitation in the data with possible deviations from the model assumptions (GLMM) for this outcome. The robustness of the findings was tested with an alternative model (GLMM PQL) with somewhat deviating results (near significant vs. significant findings, respectively). Although this overall indicates an interaction effect in favor of the self-help book, it also suggests the need for a larger patient sample to validate the findings.

## Conclusion

Sleep problems are very common in the general population and most people who seek medical attention receive treatment with medications [1]. There is a need for low-threshold interventions and treatment alternatives that are cheap and easily accessible to reduce sleep problems and the use of hypnotics. This study showed that a self-help book reduced use of hypnotics and reduced anxiety symptoms, compared with a control group. The participants in the self-help book group also reported improved sleep. However, the improvements were small, and larger studies with low-threshold interventions are warranted. The sleep hygiene advice had some positive effects on sleep problems, but the proportion using hypnotics daily increased slightly, as did the proportion reporting anxiety.
